# Epilepsy in autism spectrum disorder: examining prevalence and associated factors in a large cross-sectional study

**DOI:** 10.1186/s11689-026-09711-2

**Published:** 2026-06-13

**Authors:** Fouad Alshaban, Éric Fombonne, Iman Ghazal, Fatema Al-Faraj, Sarah Aqel, I. Richard Thompson

**Affiliations:** 1 Neurological Disorders Research Center, Qatar Biomedical Research Institute, Hamad Bin Khalifa University, Doha, Qatar; 2 College of Health and Life Sciences, Hamad Bin Khalifa University, Doha, Qatar; 3 Department of Psychiatry, School of Medicine, Oregon Health & Science University, Portland, OR USA; 4 The North East and Yorkshire NHS Genomic Laboratory Hub, Leeds, England

**Keywords:** Epilepsy, Autism Spectrum Disorder, Comorbidity, Neurodevelopmental Disorder, Qatar

## Abstract

**Background:**

Epilepsy and Autism Spectrum Disorder (ASD) frequently co-occur, yet the prevalence and factors associated with epilepsy within autistic individuals remain insufficiently defined. This study aimed to determine the prevalence of epilepsy and examine demographic, developmental, and medical correlates in a large cohort of autistic individuals in Qatar.

**Methods:**

We conducted a cross-sectional analysis of 1,257 autistic individuals recruited through clinical and research channels in Qatar. Bivariate and multivariable logistic regression analyses were used to identify factors associated with epilepsy, with false discovery rate correction applied for multiple comparisons.

**Results:**

Epilepsy was identified in 10.7% of the cohort, with affected individuals being significantly older (7.33 years) than those without epilepsy (6.59 years, *p* = 0.003). Factors associated with epilepsy included developmental delays—particularly in motor milestones such as delayed sitting (aOR = 2.954, *p* < 0.001) and walking (aOR = 3.289, *p* < 0.001)—and perinatal complications such as hypoxia (aOR = 3.188, *p* = 0.015). Behavioral features, including sleep disturbances (aOR = 5.167, *p* < 0.001) and anxiety (aOR = 2.334, *p* < 0.001), were also significantly associated.

**Conclusion:**

These findings highlight the complex interplay between developmental, behavioral, and perinatal factors associated with epilepsy within this cohort of autistic individuals and underscore the importance of systematic monitoring and multidisciplinary management to optimize outcomes in this cohort.

## Introduction

Epilepsy is characterized by recurrent unprovoked seizures caused by abnormal brain electrical activity [1]. It is one of the most common neurological disorders globally, affecting individuals across all ages and often leading to substantial cognitive, behavioral, and psychosocial consequences [2]. The condition encompasses a broad spectrum of seizure types, including focal onset, generalized onset, and unknown onset seizures, each associated with distinct neural mechanisms and clinical outcomes. Autism Spectrum Disorder (ASD) is a neurodevelopmental condition characterized by persistent differences and challenges in social communication and interaction, alongside restricted and repetitive behaviors [3]. While traditionally described using a deficit-based framework, autism is increasingly understood within a neurodiversity perspective that recognizes these characteristics as differences rather than solely impairments. The prevalence of ASD has risen significantly in recent years, with a 175% increase in autism diagnoses from 2011 to 2022, particularly among young adults [4]. ASD frequently coexists with other conditions such as epilepsy, attention deficit hyperactivity disorder (ADHD), and various genetic syndromes. Among these, epilepsy is one of the most prevalent comorbidities, with a considerable proportion of autistic individuals experiencing seizures [5, 6], suggesting shared etiological and pathophysiological mechanisms. The prevalence of epilepsy among autistic individuals has been estimated to range from 7% to 19% [7, 8]. Evidence suggests a bidirectional relationship, with autistic individuals having an increased risk of epilepsy, and vice versa [8–11]. This overlap may be explained by shared genetic and neurobiological mechanisms [12]. Genetic studies have identified multiple mutations linked to both conditions, particularly in genes regulating synaptic function and neural connectivity, which are fundamental to brain development and functioning [13]. These shared mechanisms involve pathways related to gene regulation, cellular growth, synaptic signaling, and structural maintenance, with disruptions in the excitatory–inhibitory (E/I) balance further contributing to their co-occurrence [13]. Neuroimaging studies have further revealed structural and functional brain abnormalities in individuals with both conditions, including disrupted connectivity patterns and altered neural circuitry, which may underlie their shared mechanisms [14].

Epilepsy in ASD has been reported to follow a bimodal distribution, with peaks in early childhood and adolescence, but the factors driving this pattern remain unclear [15–17]. In a large cohort study of 6,975 autistic children, Ewen et al. (2019) examined four domains of ASD severity—Intellectual Disability (ID), language impairment, core ASD symptoms, and motor dysfunction—and found that all were associated with an increased risk of epilepsy in one cohort and three in the second, with ID showing the strongest association. Similarly, a recent systematic review identified intellectual disability (ID) as the most frequently associated factor with epilepsy in autistic individuals [18]. Developmental regression has also been linked to epilepsy, suggesting that the ASD–epilepsy association may be influenced by additional markers of ASD severity beyond ID [10, 19, 20]. Other factors, including female gender, psychiatric and behavioral disorders, language impairment, and social difficulties, have also been associated with epilepsy, although findings remain inconsistent across studies [18]. Sleep disturbances are common in autistic individuals and have been associated with epilepsy, potentially increasing seizure susceptibility [20–24].

The co-occurrence of epilepsy and ASD is not merely a statistical correlation but presents a complex clinical picture that challenges caregivers, clinicians, and researchers alike. Individuals with both epilepsy and ASD often experience more severe cognitive impairments and behavioral difficulties than those with ASD alone [25]. These challenges extend beyond cognitive functioning, affecting social skills, adaptive behavior, and quality of life, making daily living more difficult and care more complex [26]. Evidence suggests that individuals with both conditions tend to have poorer developmental outcomes, higher healthcare needs, and face an elevated risk for additional comorbidities [27]. This dual diagnosis requires specialized management and tailored interventions, as standard treatments for ASD or epilepsy alone may be insufficient or inappropriate for this subgroup [28, 29]. Understanding the clinical profiles, prevalence, and factors associated with this comorbidity is crucial for early screening, diagnosis, and tailored interventions for individuals with both ASD and epilepsy. Despite progress in understanding the intersection of these conditions, significant knowledge gaps persist regarding the demographic and clinical factors influencing their co-occurrence. Variability in study methodologies, definitions of epilepsy, and sample characteristics has resulted in inconsistent prevalence estimates and conflicting findings. This study seeks to advance the understanding of epilepsy in ASD by examining its prevalence in a large sample of autistic individuals and identifying factors associated with increased epilepsy risk. Through an integrated analysis of sociodemographic, developmental, and medical factors, we aim to provide a more comprehensive understanding of epilepsy among autistic individuals, with implications for future screening, prevention, and treatment strategies tailored to this unique group. This study provides one of the largest cross-sectional analyses of epilepsy among autistic individuals in Qatar and the region.

## Methods

### Study design

This study employed a cross-sectional approach. The primary dataset was derived from two sources: (1) Our previous ASD prevalence study within Qatar [30], and (2) newly collected data from ongoing ASD research. Records from the two sources were merged and checked for duplicates using a two-step process. First, matching was performed on combinations of full name, date of birth, and (when available) medical record/ID numbers and contact phone. Second, potential matches were reviewed manually by two team members (FA & SA). When duplicates were confirmed, the most complete and most recent record was retained. Participants with missing epilepsy data were excluded. Missing epilepsy data referred to absence of clinical documentation confirming or excluding epilepsy diagnosis. From the 1,398 autistic individuals identified in the previous ASD prevalence study, 141 were excluded because epilepsy status could not be confirmed. Participants included in the study had a confirmed diagnosis of autism based on standardized clinical assessment, documented epilepsy data, and were residents of Qatar during the recruitment period. Epilepsy was coded as present only when a clinician-documented diagnosis was available in the medical record, supported by evidence such as EEG findings. Exclusion criteria included individuals without an ASD diagnosis, those with missing epilepsy data, and non-residents of Qatar.

### Data collection and instruments

Participants were invited through various channels, including social media, institutional announcements, and collaboration with developmental, behavioral, and intervention centers providing services for autistic individuals. Recruitment was conducted primarily through autism-related clinical and research services rather than epilepsy specialty clinics. Families were contacted by phone and invited to participate in our ongoing ASD studies. Prior to the appointment, families received a brochure detailing the study’s objectives and the type of data to be collected. Written informed consent was obtained from all participating families. Data were collected through structured interviews with caregivers during scheduled appointments following informed consent.

Data collected during these appointments included sociodemographic characteristics, perinatal history, developmental history, and past medical history. Standardized assessments administered as part of the study included the Social Communication Questionnaire (SCQ) [31]. Information from prior clinical records, where available, also included Vineland Adaptive Behavior Scales, Third Edition (VABS-3) [32] data. VABS-3 data were not included in the present analyses. Autism diagnoses were confirmed through standardized clinical assessments conducted by trained clinicians. Where available, the Autism Diagnostic Observation Schedule, Second Edition (ADOS-2) [33], was administered, and diagnoses were further supported through clinical evaluation and review of medical records. All assessments were administered by trained and licensed members of the research team, including clinicians and psychologists experienced in ASD research.

### Ethical approval

Ethical approval for this study was obtained from the Institutional Review Boards (IRBs) of the Qatar Biomedical Research Institute (QBRI). All procedures adhered to the ethical principles outlined in the Declaration of Helsinki and local regulatory standards. Informed consent was obtained from all participants or their legal guardians prior to their inclusion in the study. Participants were assured of their confidentiality, and all data collected were de-identified and securely stored to prevent unauthorized access.

### Statistical analysis

Statistical analyses were conducted using IBM SPSS Statistics version 29.0.1, with additional false discovery rate (FDR) correction analyses performed in R [34]. Statistical significance was initially evaluated using a two-sided p-value threshold of 0.05. Descriptive analyses were applied to summarize the sample’s demographic and clinical characteristics. The primary outcome of interest was the prevalence of epilepsy within this cohort of autistic individuals. To investigate associations between epilepsy and relevant factors, bivariate analyses were performed using chi-square tests for categorical variables.

Mean differences in age between groups with and without epilepsy were assessed using independent t-tests. Where expected frequencies were low, Fisher’s exact test was applied to ensure accurate p-values.

Logistic regression models were constructed with epilepsy as the outcome variable, including factors selected based on statistical significance in the bivariate analyses and prior literature [7, 18, 25]. Age, gender, intellectual disability, and perinatal history were entered as a priori covariates in the multivariable models, except when that variable was the exposure of interest. We used complete case (listwise) analysis per model. For each model, participants were included if they had non-missing data on the outcome and all covariates in that model. No imputation was performed. The model’s fit was assessed using the Hosmer-Lemeshow statistics. To account for multiple comparisons, false discovery rate (FDR) correction using the Benjamini–Hochberg procedure was applied to the primary analyses. Adjusted q-values were calculated and interpreted alongside the original p-values where appropriate.

## Results

In the study cohort of 1,257 participants with ASD, 135 (10.7%) had a diagnosis of epilepsy (Table 1). The mean age of participants with epilepsy was significantly higher than those without (7.33 ± 2.606 years vs. 6.59 ± 2.713 years; *p* = 0.003). Gender distribution showed no significant difference between the two groups (*p* = 0.916), while nationality demonstrated an association in the unadjusted analyses, with a higher prevalence of epilepsy among Qatari nationals compared to other groups (*p* = 0.014). Parental education was associated with epilepsy, with lower prevalence observed among children whose parents had attained college-level education (*p* = 0.001 for both). Following false discovery rate (FDR) correction, the association remained significant for maternal education. A review of perinatal and postnatal medical histories demonstrated higher frequencies of epilepsy among ASD participants exposed to maternal diseases during pregnancy (i.e., gestational diabetes, pregnancy hypertension, and anemia) in the unadjusted analyses (*p* = 0.003).

**Table 1:** Baseline Characteristics of ASD Cohort by Epilepsy Status

Variables	All		Without Epilepsy		With Epilepsy			*p*-value	FDR-adjusted q-value
	*n* = 1257		*n* = 1122	89.3%	*n* = 135		10.7%		
Sociodemographic characteristics									
Age (mean, SD)	6.67	± 2.71	6.59	± 2.713	7.33		± 2.606	**0.003**	0.0062
Gender (n, %)								0.916	0.916
Male	1009	80.3%	900	80.7%	109		80.2%		
Female	246	19.6%	220	19.3%	26		19.6%		
Nationality (n, %)								**0.014**	0.0219
Qatar	349	27.8%	300	85.96%	49		14.04%		
Other MENA	571	45.4%	510	89.32%	61		10.68%		
Non-Arab	335	26.7%	311	92.84%	24		7.16%		
Father’s age at birth (mean, SD)	34.56	± 6.459	34.56	± 6.459	34.54		± 6.493	0.977	
≥ 36 years (n, %)	382	30.4%	329	29.3%	53		39.3%	0.166	
Mother’s age at birth (mean, SD)	30.13	± 5.461	30.19	± 5.437	29.68		± 5.643	0.351	
≥ 36 years (n, %)	171	13.6%	151	13.5%	20		14.8%	0.969	
Father’s Education (n, %)								**0.001**	0.025
Primary or below	36	2.9%	30	2.7%	6		4.4%		
Secondary	212	16.9%	181	16.1%	31		23.0%		
College	744	59.2%	698	62.2%	46		34.1%		
Master’s or PhD	219	17.4%	171	15.2%	48		35.6%		
Mother’s Education (n, %)								**0.001**	0.0125
Primary or below	41	3.3%	32	2.9%	9		6.7%		
Secondary	208	16.5%	179	16.0%	29		21.5%		
College	735	58.5%	694	61.9%	41		30.4%		
Master’s or PhD	198	15.8%	144	12.8%	54		40.0%		
Consanguinity (n, %)	227	18.1%	201	17.9%	26		19.3%	0.446	0.531
Medical History									
Prematurity (< 37 weeks) (n, %)	77	6.1%	65	5.8%	12	8.9%		0.080	0.1176
Diseases during pregnancy (n, %)	214	17.0%	181	16.1%	33	24.4%		**0.003**	0.0058
Low birth weight (n, %)	97	7.7%	86	7.7%	11	8.1%		0.84	0.875
Postnatal complications (n, %)									
Any complication	214	17.0%	177	15.8%	37	27.4%		**0.001**	0.0083
Hypoxia	78	6.2%	58	5.2%	20	14.8%		**0.001**	0.0062
Jaundice	109	8.7%	90	8.0%	19	14.1%		**0.007**	0.0117
Hospital admissions during infancy (n, %)	168	13.4%	140	12.5%	28	20.7%		**0.001**	0.005
Head trauma (n, %)	26	2.1%	19	1.7%	7	5.2%		**0.003**	0.0054
ADHD (n, %)	420	33.4%	369	32.9%	51	37.8%		0.299	0.4153
Anxiety (n, %)	177	14.1%	138	12.3%	39	28.9%		**0.001**	0.0042
Aggression (n, %)	121	9.6%	105	9.4%	16	11.9%		0.353	0.4645
Sleep disturbances (n, %)	145	11.5%	101	9.0%	44	32.6%		**0.001**	0.0036
Social and behavioral parameters									
Non-verbal (n, %)	115	9.1%	97	8.6%	18	13.3%		0.77	0.837
Language delay (n, %)	343	27.3%	308	27.5%	35	25.9%		0.689	0.783
Motor delay (n, %)									
Sitting ≥ 8 months	138	11.0%	105	9.4%	33	24.4%		**0.001**	0.0031
Walking ≥ 18 months	112	8.9%	83	7.4%	29	21.5%		**0.001**	0.0028
Regression of skills (n, %)	256	20.4%	206	18.4%	50	37.0%		**0.001**	0.0025
Learning difficulties (n, %)	892	71.0%	780	69.5%	112	83.0%		**0.001**	0.0023
Intellectual disability (n, %)	142	11.3%	124	11.1%	18	13.3%		0.385	0.4812

Diseases during pregnancy include common maternal conditions such as gestational diabetes, hypertension, and anemia. p-values calculated using independent t-tests for continuous variables and chi-square tests for categorical variables. *p* < 0.05 considered statistically significant. False discovery rate (FDR) correction using the Benjamini–Hochberg procedure was additionally applied to account for multiple comparisons. Learning difficulties and intellectual disability were treated as distinct constructs. Learning difficulties refer to academic challenges, whereas intellectual disability reflects global cognitive impairment

*MENA* Middle East and North Africa, *SD* standard deviation

Likewise, hypoxia (*p* = 0.001), jaundice (*p* = 0.007), head trauma (*p* = 0.003), and hospitalization during infancy (*p* = 0.001) showed significant associations in the unadjusted analyses; however, following FDR correction, hypoxia remained among the strongest perinatal associations. Developmental and behavioral characteristics differed significantly between groups, with higher frequencies of anxiety (*p* = 0.001), sleep disturbances (*p* = 0.001), delayed sitting (*p* = 0.001), delayed walking (*p* = 0.001), regression of skills (*p* = 0.001), and learning difficulties (*p* = 0.001) among participants with epilepsy. These associations remained significant following FDR correction.

Initial (crude) logistic regression models demonstrated associations between multiple variables and epilepsy (Table 2). Age emerged as a significant variable, with each additional year associated with higher odds of epilepsy (OR = 1.085, 95% CI: 1.026–1.147, *p* = 0.004). Perinatal complications demonstrated notable associations, with individuals exposed to diseases during pregnancy showing elevated odds (OR = 1.912, 95% CI: 1.242–2.945, *p* = 0.003), and those with histories of hypoxia and jaundice demonstrating three-fold (OR = 3.503, 95% CI: 2.025–6.061, *p* < 0.001) and two-fold (OR = 2.057, 95% CI: 1.204–3.514, *p* = 0.008) higher odds of epilepsy, respectively. Behavioral and developmental delays also demonstrated significant associations; children with anxiety had nearly three times the odds of epilepsy (OR = 2.897, 95% CI: 1.917–4.376, *p* < 0.001), and those with sleep disturbances exhibited even higher odds (OR = 4.888, 95% CI: 3.231–7.394, *p* < 0.001). Developmental delays in sitting (OR = 3.858, 95% CI: 2.453–6.067, *p* < 0.001) and walking (OR = 4.164, 95% CI: 2.582–6.714, *p* < 0.001), along with skill regression (OR = 3.723, 95% CI: 2.481–5.588, *p* < 0.001) and learning difficulties (OR = 2.181, 95% CI: 1.344–3.539, *p* = 0.002), were also significantly associated with epilepsy.

**Table 2:** Crude and Adjusted Logistic Regression Analyses of Factors Associated with Epilepsy in Autistic Individuals

Variable	Crude OR (95% CI)	*p*-value	Adjusted OR (95% CI)	Adjusted *p*	FDR-adjusted q-value
Age (years)	1.085 (1.026–1.147)	0.004	1.074 (1.011–1.141)	**0.021**	0.0443
Nationality (Qatar)	1.578 (1.084–2.297)	0.017	1.482 (0.977–2.249)	0.064	0.1013
Nationality (non-Arabs)	0.568 (0.359–0.901)	0.016	0.677 (0.409–1.122)	0.13	0.1453
Father’s Education (College and above)	0.330 (0.131–0.832)	0.019	0.348 (0.131–0.929)	**0.035**	0.0665
Mother’s Education (College and above)	0.210 (0.094–0.469)	< 0.001	0.272 (0.105–0.703)	**0.007**	0.019
Diseases during pregnancy	1.912 (1.242–2.945)	0.003	1.633 (1.016–2.626)	**0.043**	0.0743
Low birth weight (< 2500 g)	2.544 (1.271–5.093)	0.008	1.897 (0.860–4.181)	0.113	0.1534
Postnatal complications	2.259 (1.485–3.437)	< 0.001	0.795 (0.303–2.087)	0.641	0.6766
Hypoxia	3.503 (2.025–6.061)	< 0.001	3.188 (1.253–8.115)	**0.015**	0.0356
Jaundice	2.057 (1.204–3.514)	0.008	2.089 (0.823–5.303)	0.121	0.1533
Requiring hospital admission during infancy	2.078 (1.314–3.288)	0.002	1.515 (0.907–2.531)	0.112	0.1637
Head trauma	3.489 (1.436–8.476)	0.006	2.185 (0.813–5.875)	0.121	0.1437
Anxiety	2.897 (1.917–4.376)	< 0.001	2.334 (1.456–3.740)	**< 0.001**	0.0095
Sleep Disturbances	4.888 (3.231–7.394)	< 0.001	5.167 (3.204–8.333)	**< 0.001**	0.0048
Delayed sitting	3.858 (2.453–6.067)	< 0.001	2.954 (1.788–4.879)	**< 0.001**	0.0032
Delayed walking	4.164 (2.582–6.714)	< 0.001	3.289 (1.930–5.607)	**< 0.001**	0.0024
Regression of skills	3.723 (2.481–5.588)	< 0.001	3.981 (2.511–6.311)	**< 0.001**	0.0019
Learning difficulties	2.181 (1.344–3.539)	0.002	2.513 (1.440–4.385)	**0.001**	0.0032
Intellectual disability	1.265 (0.743–2.153)	0.386	1.087 (0.610–1.939)	0.777	0.777

Each variable was examined in an individual multivariable model adjusted for child age, gender, perinatal complications (diseases during pregnancy, hypoxia, jaundice, and admissions during infancy), and intellectual disability. Hypoxia was defined based on caregiver report of oxygen deprivation at birth or documented clinical diagnosis in medical records. False discovery rate (FDR) correction using the Benjamini–Hochberg procedure was applied to adjusted model p-values to account for multiple comparisons. q-values < 0.05 were considered statistically significant after correction

*OR* odds ratio, *CI* confidence interval

Logistic regression models adjusted for age, gender, intellectual disability, and perinatal complications (diseases during pregnancy, hypoxia, jaundice, and hospital admissions during infancy) identified several variables associated with epilepsy **(**Table 2**)**. Following FDR correction, the strongest associations remained statistically significant. Older age remained associated with slightly higher odds of epilepsy following FDR correction (aOR = 1.074, 95% CI: 1.011–1.141, *p* = 0.021). Higher maternal education remained associated with lower odds of epilepsy following FDR correction (aOR = 0.272, 95% CI: 0.105–0.703, *p* = 0.007), whereas the association with paternal education did not remain statistically significant after correction.

Among perinatal factors, hypoxia remained significantly associated with epilepsy following FDR correction (aOR = 3.188, 95% CI: 1.253–8.115, *p* = 0.015). Behavioral and developmental characteristics remained robustly associated with epilepsy following FDR correction, including anxiety (aOR = 2.334, 95% CI: 1.456–3.740, *p* < 0.001), sleep disturbances (aOR = 5.167, 95% CI: 3.204–8.333, *p* < 0.001), delays in sitting (aOR = 2.954, 95% CI: 1.788–4.879, *p* < 0.001) and walking (aOR = 3.289, 95% CI: 1.930–5.607, *p* < 0.001), regression of skills (aOR = 3.981, 95% CI: 2.511–6.311, *p* < 0.001), and learning difficulties (aOR = 2.513, 95% CI: 1.440–4.385, *p* = 0.001).

## Discussion

This study found a 10.7% prevalence of epilepsy among autistic individuals, aligning with previously reported rates ranging from 7% to 19% in autistic individuals [7]. In the general population, the lifetime prevalence of epilepsy is ~ 0.8% (7.60 per 1,000) [35]. The substantially higher prevalence observed among autistic individuals relative to the general population supports the importance of systematic epilepsy surveillance as a routine component of ASD care. Consistent with prior reports, older age was associated with a higher risk of epilepsy in ASD, aligning with evidence that epilepsy prevalence increases from childhood into adolescence and young adulthood [8]. Beyond a simple age gradient, several mechanisms may contribute.

First, older participants may simply have had more time for seizure onset to occur during development. Separately, delayed recognition of seizures, particularly non-motor seizures, may contribute to epilepsy being diagnosed later in childhood or adolescence, as reported in pediatric epilepsy studies [36, 37]. Second, hormonal changes during puberty and the associated synaptic remodeling have been linked to shifts in seizure susceptibility, a pattern observed in ASD cohorts where adolescent-onset seizures are relatively common [38]. Third, certain forms of epilepsy, particularly non-motor seizures, are often under-recognized, leading to delayed diagnosis [39]. Collectively, these factors may explain the higher prevalence of epilepsy observed among older autistic individuals.

Several studies report a higher risk of epilepsy in autistic females than males, though findings are mixed [8, 25, 40]. In our cohort, however, gender was not a significant predictor of epilepsy. The gender distribution was comparable between the epilepsy (80.2% male, 19.6% female) and non-epilepsy groups (80.7% male, 19.3% female), aligning with the typical male predominance in ASD. Previous studies have suggested that the higher prevalence of comorbid intellectual disability among females with ASD may contribute to the observed gender differences in epilepsy risk, indicating that cognitive ability rather than gender itself may play a more significant role in this association [8, 41]. In contrast to prior reports [10, 18], our analysis did not identify a significant association between intellectual disability and epilepsy. This difference may reflect population or sampling characteristics unique to our cohort, emphasizing the importance of examining this relationship across diverse autistic individuals.

Higher parental education was associated with lower odds of epilepsy in ASD. This pattern is consistent with reports linking parental education to greater health literacy, earlier access to care, and timelier intervention, although unmeasured socioeconomic factors may still influence this association [42, 43]. Medical factors such as neonatal hypoxia and jaundice were also prevalent among participants with epilepsy. These patterns suggest possible early neurological compromise. However, in adjusted analyses, only neonatal hypoxia remained associated with higher odds of epilepsy, whereas the association with jaundice did not persist. Biologically, perinatal hypoxia may contribute to cortical injury and excitatory–inhibitory imbalance, potentially increasing seizure susceptibility in vulnerable children [44]. By contrast, evidence linking neonatal jaundice to later seizures is heterogeneous [45, 46]. The attenuation after adjustment may reflect milder or promptly treated neonatal illness (e.g., timely phototherapy) and other measured perinatal factors that account for the crude association.

Moreover, sleep disturbances and anxiety showed positive associations with epilepsy. These findings are consistent with literature noting the bidirectional relationship between epilepsy and behavioral symptoms, where seizures may aggravate existing issues, potentially reinforcing a difficult clinical cycle in ASD [20]. Similarly, Viscidi et al. [8] reported that autistic individuals with epilepsy exhibited more pronounced behavioral impairments than those without epilepsy. Delays in motor milestones and skill regression were also more common among participants with epilepsy, aligning with previous findings that developmental delays, particularly in motor function, may indicate early neurodevelopmental vulnerability and increase susceptibility to epilepsy [18]. However, evidence on regression is mixed, with some studies finding an association [16] and others not [8]. It is possible that subclinical epileptic activity may be associated with behavioral and developmental features rather than these features predisposing individuals to epilepsy. Alternatively, shared neurodevelopmental mechanisms may underlie both conditions, creating a bidirectional relationship in which each influences and potentially amplifies the other.

In this study, several factors remained significantly associated with epilepsy after multivariable adjustment and FDR correction, including hypoxia, developmental delays, sleep disturbances, anxiety, regression of skills, and learning difficulties. These findings suggest that the strongest observed associations were robust even after accounting for multiple statistical comparisons.

### Strengths, limitations and future directions

This study has several notable strengths. First, the large sample size of 1,257 autistic individuals enhances the robustness of our findings, allowing for more reliable estimates of epilepsy prevalence and associated factors in autistic individuals with epilepsy. The use of multiple data sources strengthens the diversity of the sample and minimizes potential biases inherent in single-source datasets. Additionally, the detailed analysis of sociodemographic, medical, and behavioral variables provides a comprehensive assessment of factors associated with epilepsy within ASD, contributing to a more nuanced understanding of this comorbidity. However, the study also has limitations. Its cross-sectional design precludes causal inferences regarding the observed associations between epilepsy and various associated factors. This design also relies on historical data, which may be prone to recall bias. Unmeasured factors such as genetic markers could also play a role in epilepsy risk and were not examined. Although multiple comparison correction was applied in the revised analyses, the possibility of residual type I error cannot be completely excluded given the exploratory nature of several investigated associations.

Our findings are based on data from a specific population, and while the results align with existing literature, replication in other populations and settings would strengthen the generalizability of the conclusions. Future research should prioritize prospective, longitudinal studies to clarify temporal relationships to distinguish whether developmental and behavioral characteristics precede, accompany, or result from epilepsy. Such designs, integrating genetic, neuroimaging, and environmental data, will be crucial for elucidating underlying mechanisms and informing evidence-based clinical strategies. Importantly, the sample was not population-representative, as participants were recruited through clinical and research channels. Therefore, prevalence estimates should be interpreted within the context of this cohort and not generalized to the broader autistic population.

## Conclusion

This study highlights key developmental, behavioral, and perinatal factors associated with epilepsy within this cohort of autistic individuals. Factors that remained robustly associated with epilepsy following multivariable adjustment and FDR correction included hypoxia, developmental delays, sleep disturbances, anxiety, regression of skills, and learning difficulties. With a prevalence of 10.7%, epilepsy remains a significant comorbidity in ASD, demanding vigilant, tailored care that addresses not only seizure management but also the broader neurodevelopmental and psychosocial context of each individual. Our findings highlight specific developmental, behavioral, and perinatal factors that may inform earlier recognition and more targeted clinical monitoring of epilepsy in autistic individuals. Recognizing these nuanced clinical profiles paves the way for a more personalized approach to ASD care, fostering improved outcomes and quality of life. As we deepen our understanding of the overlapping mechanisms driving both ASD and epilepsy, there lies a critical opportunity to develop evidence-based strategies that can inform clinical practice and future research directions for individuals affected by these intersecting conditions.

## Data Availability

The datasets generated and/or analysed during the current study are not publicly available due to the confidentiality and sensitivity of the data but are available from the corresponding author on reasonable request.

## Abbreviations

ADHD: Attention-Deficit/Hyperactivity Disorder
ADOS-2: Autism Diagnostic Observation Schedule, Second Edition
aOR: Adjusted Odds Ratio
ASD: Autism Spectrum Disorder
CI: Confidence Interval
EEG: Electroencephalography
ID: Intellectual Disability
IRB: Institutional Review Board
MENA: Middle East and North Africa
OSA: Obstructive Sleep Apnea
OR: Odds Ratio
QBRI: Qatar Biomedical Research Institute
SCQ: Social Communication Questionnaire
SD: Standard Deviation
VABS-3: Vineland Adaptive Behavior .Scales, Third Edition
